# Supplementary material for: Genetic and Metabolic Factors of Familial Dysbetalipoproteinemia Phenotype: Insights from a Cross-Sectional Study
Source: Int J Mol Sci. 2025 Jul 30;26(15):7376. doi: 10.3390/ijms26157376 (PMC12347968; doi:10.3390/ijms26157376)
Supplement: Supplementary file 1 [file ijms-26-07376-s001.zip › TableS1_Supplementary Material.pdf]

Supplementary Table S1. Associations between FD phenotype-related factors and TG levels obtained by univariable linear regression analysis.

Predictor (observations, *n*)	β coefficient (SE)	95% CI	*p*-Value	R^2	Residual SE
Polygenic HTG, per 1 percentile (68)	0.04 (0.02)	-0.01; 0.08	0.1	0.04	5.21
Variants in other lipid-related genes (71)	6.40 (1.70)	3.01–9.79	<0.001 **	0.17	4.76
Age, per 1 year (71)	-0.001 (0.05)	-0.10; 0.10	0.988	0	5.23
Sex, female (71)	0.27 (1.25)	-2.22; 2.76	0.829	0.001	5.23
BMI, per kg/m² (71)	-0.06 (0.13)	-0.32; 0.20	0.635	0.003	5.22
Obesity (71)	0.06 (1.26)	-2.44; 2.57	0.961	0	5.23
WC, per 1 cm (58)	0.01 (0.05)	-0.10; 0.12	0.836	0.001	5.25
WHtR, per 0.1 unit (58)	-1.20 (8.58)	-18.38; 15.99	0.889	0	5.26
Metabolic syndrome components (71)	0.79 (1.27)	-1.74; 3.31	0.537	0.01	5.21
Glucose metabolism disorders (71)	3.41 (1.67)	0.09–6.73	0.04 *	0.06	5.08
Hypothyroidism (69)	0.92 (2.45)	-3.97; 5.80	0.709	0.002	5.27

** p <0.001, * p <0.05. The table shows the regression coefficients (β), standard errors (SE), 95% confidence intervals (CI), *p*-values, multiple R-squared values (R^2), and residual standard errors for each predictor. BMI—body mass index; CI—confidence interval; HTG—hypertriglyceridemia; R^2—multiple R-squared; SE—standard errors; WC—waist circumference; WHtR—waist-to-height ratio.
